# Increased Resting-State Perfusion after Repeated Encoding Is Related to Later Retrieval of Declarative Associative Memories

**DOI:** 10.1371/journal.pone.0019985

**Published:** 2011-05-12

**Authors:** Georg Groen, Alexander N. Sokolov, Christina Jonas, Robert Roebling, Manfred Spitzer

**Affiliations:** 1 Section for Neuropsychology and Functional Imaging, Department of Psychiatry, Ulm University, Ulm, Germany; 2 Centre for Ophthalmology, Low Vision Clinic and Research Laboratory, Eberhard Karls University of Tübingen, Tübingen, Germany; Beijing Normal University, Beijing, China

## Abstract

Electrophysiological studies in animals have shown coordinated reactivation of neuronal ensembles during a restricted time period of behavioral inactivity that immediately followed active encoding. In the present study we directly investigated off-line processing of associative memory formation in the human brain. Subjects' regional cerebral blood flow (rCBF) as a surrogate marker of neural activity during rest was measured by MR-based perfusion imaging in a sample of 14 healthy male subjects prior to (Pre2) and after (Post) extensive learning of 24 face-name associations within a selective reminding task (SR). Results demonstrated significant Post-Pre2 rCBF increases in hippocampal and temporal lobe regions, while in a control comparison of two perfusion scans with no learning task in-between (Pre2-Pre1) no differences in rCBF emerged. Post perfusion scanning was followed by a surprise cued associative recall task from which two types of correctly retrieved names were obtained: older names already correctly retrieved at least once during one of the SR blocks, and recent names acquired during the last SR block immediately prior to the Post scan. In the anterior hippocampus individual perfusion increases were correlated with both correct retrievals of older and recent names. By contrast, older but not recently learned names showed a significant correlation with perfusion increases in the left lateral temporal cortex known to be associated with long-term memory. Recent, but not older names were correlated with dopaminergic midbrain structures reported to contribute to the persistence of memory traces for novel information. Although the direct investigation of off-line memory processing did not permit concomitant experimental control, neither intentional rehearsal, nor substantial variations in subjects' states of alertness appear to contribute to present results. We suggest that the observed rCBF increases might reflect processes that possibly contribute to the long-term persistence of memory traces.

## Introduction

Animal studies strongly support offline memory processing to take place during phases of behavioral inactivity (either sleep or quiet wakefulness) that follow the initial experience [1]–[7]. These studies demonstrated that after an experience, parts of the brain show correlated patterns of neural activity resembling those that had already occurred during the corresponding experience. This observation has been interpreted as reactivation of memory traces and theoretical concepts originating with Marr [8]–[12] suggest that spontaneous reactivation of memory traces after their initial encoding subserves a supplementary training function. Thereby new memories can undergo gradual changes from an initially fragile state into more permanent and stable neural representations, rendering them resistant to interference and easing their availability for latter intentional recollection. From a systems perspective, this reactivation is provided by interplay of neural activities between regions of the cortex (e.g. the hippocampus and related associational structures) that were already engaged during initial encoding. Through such repeated subsequent replay of patterns of neural activity in a hippocampal-cortical network the specific memory trace is efficiently encoded and transformed into long-term memory.

While this off-line memory processing in the wakeful brain has been directly observed in animal physiology, evidence for such processes in the human brain is scarce. In the domain of perceptuomotor learning two recent studies investigated neural activity before and after a motor learning task. These studies used either resting-state low-frequency BOLD fluctuations [13], or compared averaged blocks of a rest condition against blocks of an active condition during a simple motor task [14]. In both studies a preceding task modulated neural signaling afterwards supporting reactivation of task related experiences. Moreover, in the domain of associative memory a very recent study [15] compared off-line functional connectivity between the hippocampus and a portion of the lateral occipital complex before and after an associative encoding task observing enhanced functional connectivity during rest after learning.

**Figure 1:**
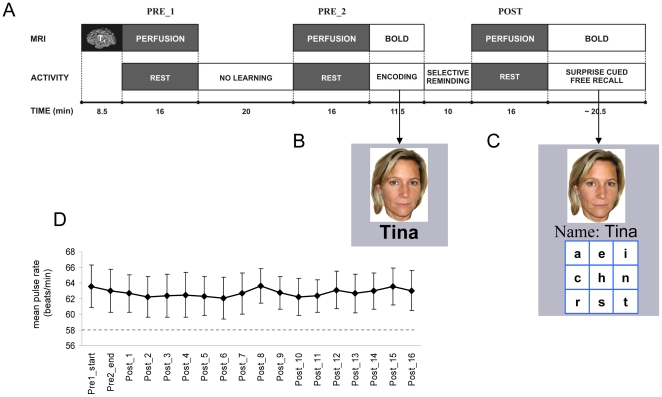
Experimental design and behavioral data. (A) Perfusion images were acquired during experimental blocks PRE 1, PRE 2, and POST where participants should relax and rest in quiet wakefulness in the MR scanner. Between PRE 2 and POST initial encoding of face-name associations took place under event-related BOLD acquisition, followed by blocks of selective reminding without scanning. BOLD images were again acquired after the last perfusion scan during the final surprise associative cued recall task. (B). An example of the face-name associations each presented for 3 seconds during encoding. (C). An example of one trial of cued associative free recall. Upon presentation of the cue subjects could type the corresponding name using a 3-by-3 keyboard. (D). Mean heart rates (error bars reflect standard error of the mean) sampled at the start of Pre1 (2nd minute), at the end of Pre2 (last minute) and at each minute of the 16 minutes lasting perfusion scan after the learning task inserted (Post). The dashed line shows the critical mean frequency of 58 bpm (see Methods section).

In order to further investigate off-line memory processing following the encoding of associative memories, we also adopted a pre-post learning study design from previous electrophysiological studies in experimental animals (e.g. [1]). Encoding and selective reminding (SR) of face-name associations was placed between two sessions of measuring subjects' neural activity at rest for an interval of 16 minutes each (Figure 1, Pre2 and Post). In contrast to previous studies, neural activity was measured using continuous arterial spin labelling (CASL) [16]. CASL provides a measure of the neural metabolism in terms of regional cerebral blood flow (rCBF) which most likely reflects the energetically demanding synaptic activity in a specific brain region [17], [16]. Changes of perfusion can therefore be regarded as a surrogate marker of altered neuronal activity. Consequently, it was predicted that brain perfusion during the sixteen minutes following the extensive learning of face-name associations should significantly change when compared against the same time interval of brain perfusion that preceded the learning phase. As a control another 16 minute perfusion scan (Figure 1; Pre1) was part of the design and allowed us to compare brain activities at rest with no learning task in-between. Furthermore, the Post perfusion scan was followed by a surprise cued associative recall task to assess learning success. Due to the selective reminding procedure correctly retrieved names obtained from the final cued recall task could be categorized into two categories: correct older and correct recent names. Correct older names were operationally defined as those names that had already been correctly retrieved at least once during one of the SR blocks. Complementary, correct recent names had not been correctly retrieved during any of the SR blocks, but were successfully acquired (in terms of correct final recall) during the last SR block immediately prior to the Post perfusion scan. Individual expressions of both response categories served as a set of two different regressors to further explore whether the putative Post-Pre2 perfusion changes were functionally associated with off-line memory processing.

## Results

### Monitoring states of alertness

For each subject the individual heart rate was binned in one minute time-slots at various index time points across the entire experimental session. For comparative purposes, the second minute of Pre1 served as the individual baseline. Neither in the comparison with the last minute of Pre2 nor in any of the 16 time points of the Post perfusion scan individual heart rates exceeded the chosen cut-off of 11% (see Methods), which would be indicative of a substantial variation of each subject's state of alertness. A summary of mean pulse rates averaged across subjects is depicted in Figure 1D.

### Behavioral Performance

**Figure 2:**
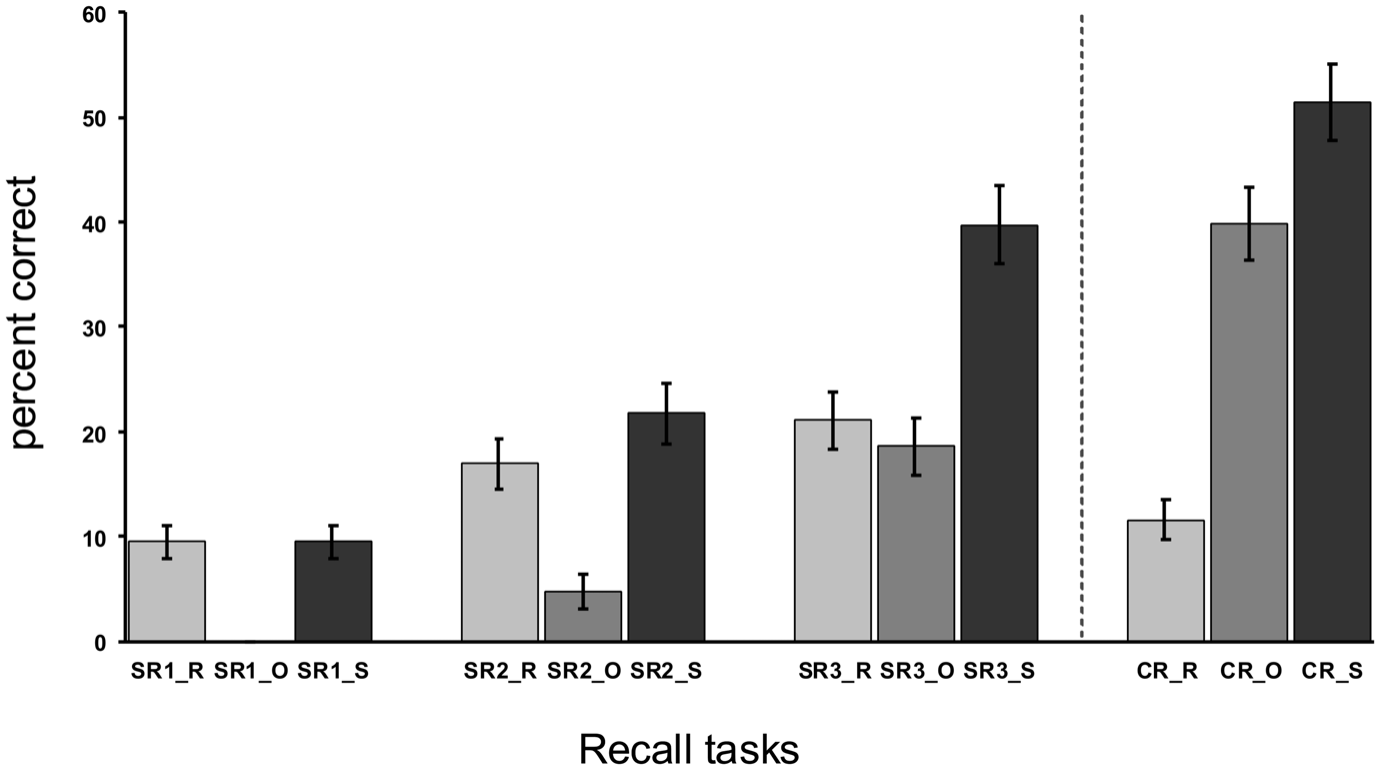
Memory performance in three consecutive repetitions of selective reminding blocks (SR1–SR3) and during cued associative free recall (CR). Subjects had to actively recall the corresponding name upon appearance of the face. Light grey bars reflect the proportions of correctly recalled names encoded during the preceding selective reminding block (_R: correct recent names; in case of SR 1 the preceding learning task was initial encoding). Middle grey bars reflect proportions of names that had already been correctly recalled at least once during preceding SR blocks (_O: correct older names). Dark grey bars illustrate overall memory performance (_S: sum of percent correct). Error bars refer to the standard error of the mean. The grey dashed line illustrates the Post perfusion scan that took place between the very last SR block (SR3) and the CR task. For overall memory performance trend tests revealed that the increase in performance followed a highly significant linear trend (F(1,13) = 136.42; p<0.001 ), with no other trends yielding significant fits. Also for correct older names the increase from the second SR block (SR2_O) up to and including the CR task (CR_O) was significantly linear (F(1,13) = 116.03, p<0.001); no other trends emerged. For correct recent names there was no significant linear trend (F(1,13) = 1.89; p = 0.192). Changes in performance could rather be fitted by a significant quadratic term (F(1,13) = 58.02; p = 0.005).

Only correct responses in the selective reminding and the cued associative recall tasks were analyzed, after subdivision in older and recent responses (see Methods section). A summary of these proportions is depicted in Figure 2 and summary statistics is tabulated in Table 1. Overall memory performance significantly increased from the first selective reminding block (SR1) up to and including the CR task. Post-hoc tests revealed that there was still a significant difference between the third selective reminding block (SR3) and the CR task although the rate of correct recent names significantly decreased from SR 3 to CR (see Table 1 for results of post-hoc testing). Comparing proportions of correct retrievals within the CR task, the difference between older and recent names was significant (t(14) = 6.50; p<0.001). Moreover, comparison of response times for each response category during the CR task yielded a significant result (t(13) = 2.29; p = 0.039). Average response times for older correctly retrieved names (mean: 4.56 sec.; SD: 1.02) were faster than for correct recent names (mean: 5.67 sec: SD: 2.05). Therefore both sub-types of correct responses were considered separately as regressors for correlations with perfusion changes (see below).

**Table 1:** Statistical analysis of memory performance during recall tasks.

Type of analysis	Main effect	Post hoc tests
Overall memory performance(including SR1_S to CR_S)	F(3,39) = 72.32;GG-ε = 0.727;p<0.001	SR1_S<SR2_S<SR3_S<CR_S
Recent correct names(including SR1_R to CR_R)	F(3,39) = 5.54;GG-ε = 0.874;p = 0.005	SR3_R>CR_RSR3_R>SR1_R
Older correct names(including SR2_O to CR_O)	F(2,26) = 78.64;GG-ε = 0.869;p<0.001	SR2_O<SR3_O<CR_O

Post hoc tests refer to Newman-Keuls Test; <> denotes significant differences at a nominal level of significance of p<0.05;  = denotes non-significant differences; in case of overall memory performance and older correct names transitivity of post hoc testing holds (e.g. SR1_S<CR_S); for recent correct names no other significant differences than those tabulated were observed.

### Imaging results

#### Comparisons of rCBF changes

**Figure 3:**
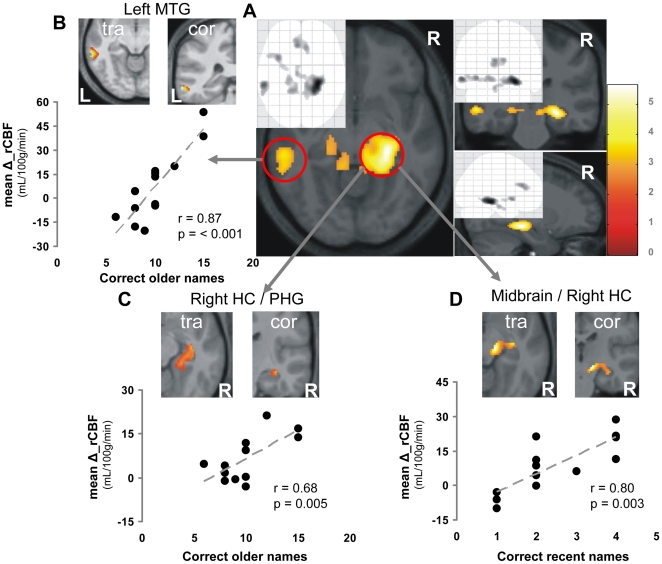
Results from perfusion imaging. (A) Brain regions with significant (p<0.005 at the voxel) perfusion increases from Pre2 to Post (mean Δ_rCBF in units of mL/100 g tissue/min). Significant clusters (p<0.05) were superimposed on different slices of a standardized T1 weighted image calculated as average of individually normalized T1 images (MNI space). The transversal slice shows the right hippocampal region (parahippocampal gyrus and hippocampus proper), left parahippocampal gyrus (PHG), left hippocampus (HC) and left middle temporal gyrus (MTG). The coronal view shows the bilateral hippocampus and left middle temporal gyrus. The sagittal slice shows the right hippocampus posteriorly extending into the parahippocampal gyrus. Insets are glass brains to demonstrate all brain regions with significant Post-Pre2 perfusion increases. Anatomical locations are summarized in Table 2. Only voxels of clusters marked by red circles revealed significant positive correlations. (B) Scatterplot depicting the maximum significant positive correlation (peak voxel) between individual Post-Pre2 perfusion increases and individual numbers of correct older names (already retrieved at least once during one of the SR blocks). In the left MTG no significant correlations were observed for correct recent names (successfully encoded just during the very last selective reminding block (SR3), but not in any of the preceding SR blocks (SR1, SR2)). (C) Part of the right hippocampal cluster where perfusion increases were correlated with correct older names. (D). The correlation with correct recent items also produced a significant positive correlation in the right hippocampus, but more anterior to that observed for correct older names. Medially, the correlation with correct recent items extended into the midbrain comprising the right substantia nigra (scatterplot from SN's peak voxel), where perfusion increases were not correlated with correct older names.

Comparison (one-tailed t-contrast) of mean regional cerebral blood flow (rCBF) before and after the learning task (Post-Pre2) revealed significant increases of rCBF in the left and especially in the right hippocampal region (parahippocampal gyrus and hippocampus proper; Figure 3A) and the left middle temporal gyrus (Figure 3A). Additional clusters were observed in the left and right lingual gyri, the left insular region, and the anterior cingulate. A detailed description of significant anatomical locations is given in Table 2. For further control against random changes over time both Pre-sessions were contrasted with a direction that tested for regional CBF increases during Pre 2 (Pre2-Pre1). A whole brain analysis (same significance thresholds as above) did not reveal any significant differences. In a second test the outcome of the Post-Pre2 contrast was used as an inclusive functional mask to test Pre2-Pre1 differences for significance at a very liberal threshold of p<0.1 (uncorrected). Again, no significant results were observed.

**Table 2:** Brain regions with significant rCBF differences in the contrast Post-Pre2.

	*MNI coordinates (mm)*				
*Anatomical Region*	*X*	*y*	*z*	*Z score*	*BA*
Right Hippocampus*	26	−28	−10	5.03	
Right Parahippocampal Gyrus*	26	−36	−10	4.59	36
Right Lingual Gyrus	20	−44	−8	4.13	30
Midbrain (SN/VTA complex)	14	−22	−10	3.13	
Left Middle Temporal Gyrus*	−58	−32	−6	4.09	21
Left Insula	−34	12	−6	3.69	13
Left Anterior Cingulate Gyrus	−6	32	20	3.64	24
Right Anterior Cingulate Gyrus	8	28	22	3.41	24
Left Lingual Gyrus	−14	−40	−6	3.41	30
Left Hippocampus	−20	−28	−8	3.12	

MNI: Montreal Neurological Institute; BA: Brodmann Area; Z scores of peak voxels refer to standard normal distribution and were derived from an one-tailed t contrast on the difference in mean rCBF between the POST and PRE 2 perfusion scans with voxel-level significance of p<0.005, uncorrected, and cluster-level significance at p<0.05 (corresponding to 184 contiguously significant voxels).

*: denotes anatomical locations with peak voxels also surviving a false discovery rate correction at p<0.05.

### Correlation analyses

To test whether Post-Pre2 perfusion changes were correlated with individual memory performances during the cued associative recall task, two separate regression analyses were computed for recent and older correct responses. These computations were locally constrained to those voxels that already had demonstrated significant increases of rCBF between Pre2 and Post (Table 2). Significant positive correlations between rCBF increases and correct *older* responses were locally confined to the right hippocampal region (RHC) (peak voxel at 34 −20 −12; Pearson's correlation coefficient averaged across 173 significant voxels: *r_avg_ = 0.56*; *p = 0.022*; *r_min_ = 0.48*, *p = 0.047*; *r_max_ = 0.68*, *p = 0.005*; Figure 3C) and the left middle temporal gyrus (LTG) (peak voxel at −62, −36 −10; Pearson's correlation coefficient averaged across 82 significant voxels: *r_avg_ = 0.68*; *p = 0.005*; *r_min_ = 0.48*, *p = 0.047*; *r_max_ = 0.87*, *p<0.001*; Figure 3B). In both clusters, Pre2-to-Post rCBF increases were also tested for correlations with individual numbers of correct recent items (RHC: r*_avg_* = 0.32; p = 0.138; LTG: r*_avg_* = 0.31, p = 0.153) which did not produce significant findings. Statistical comparisons between correlations for either region revealed that the average correlation coefficients for older correct responses were significantly greater than for correct recent items (RHC: z = 2.43, p = 0.030; LTG: z = 2.79; p = 0.010; both p-values already Bonferroni-adjusted).

For correct *recent* responses the only cluster of significant voxels (n = 176) bearing a positive correlation was located in the right anterior hippocampal region medially reaching into midbrain structures (peak voxel at: 12, −24, −10 corresponding to the substantia nigra; average correlation across 85 significant voxels *r_avg_ = 0.61*; *p = 0.012*; *r_min_ = 0.48*, *p = 0.047*; *r_max_ = 0.80*, *p = 0.003*; Figure 3D). Correlating perfusion increases in this midbrain segment with older correct items did not reveal a significant correlation. (r*_avg_* = 0.27; p = 0.184). The difference between averaged correlation coefficients was significant (z = 2.42; p = 0.031, adjusted). The right hippocampal cluster (peak voxel at: 34, −18, −12; average correlation across 39 significant voxels *r_avg_ = 0.59*; *p = 0.016*; *r_min_ = 0.48*, *p = 0.047*; *r_max_ = 0.79*, *p = 0.004*; Figure 3D) demonstrated some anatomical overlap with the one obtained by the positive correlation with older correct responses (see Figure S1 of Supporting Information). Accordingly, although weaker, voxels in this section were also significantly positively correlated with correct older correct names (*r_avg_* = 0.51; p = 0.035), and the difference between both averaged correlation coefficients was not significant (z = 0.43; p = 0.33, uncorrected).

All observations reported above remained when data were re-analyzed using a smaller smoothing kernel of 6 mm full width of half maximum during preprocessing (see Table S1 and Figure S2 of Supporting Information for detailed results).

## Discussion

In the present study we directly investigated off-line memory processing immediately after repeated and extensive learning of a set of face-name associations. Learning took place between two perfusion scans lasting 16 minutes each for the measurement of regional cerebral blood flow (rCBF) before (Pre2) and after (Post) learning. Contrasting both sessions (Post-Pre2) revealed significant increases in rCBF in the bilateral anterior hippocampus, the left middle temporal gyrus, bilateral lingual gyrus and the anterior cingulate. A contrast comparing two consecutive perfusion scans with no encoding in-between (Pre2-Pre1) did not reveal any significant changes in perfusion. This was further confirmed even at substantially lowered significance thresholds with the Post to Pre2 changes serving as an inclusive mask.

### A dissociating pattern of brain-behavior relationships to support functional relevance of perfusion changes

To further explore the functional relevance of these perfusion increases individual scores of correctly retrieved names during cued associative recall following the Post perfusion scan were correlated with individual Post-Pre2 perfusion changes. Due to the selective reminding task before the Post perfusion scan correct name retrievals were subdivided into two response categories: Correct older names that had already been correctly recalled at least once during the preceding selective reminding blocks, and correct recent names that had been successfully encoded just during the selective reminding block that preceded the post learning perfusion scanning. Both regressors showed significant differences regarding frequency and response times, which we take as evidence that they reflect qualitatively and/or quantitatively different memory traces.

The correlation analysis led to two interesting observations. First, not all brain regions with increased rCBF showed a significant correlation with performance. This questions their functional relevance in the service of off-line memory processing. Secondly, for the right hippocampal region and adjacent midbrain structures, as well as for the left middle temporal gyrus, the regressors yielded significant and differential results. The rate of correct retrievals of older names was correlated with perfusion increases in the right anterior hippocampus posteriorly extending into the anterior parahippocampal gyrus. Additionally, older (and supposedly already more deeply encoded items) led to significant positive correlations in the left middle temporal gyrus where correct recent items did not yield significant correlation coefficients. This dissociation is in line with previous evidence generally linking the lateral temporal cortices with long-term memory [18]–[25]. Our data therefore support the assumption that the memory traces of older items have already been established within this region, which may have caused the shorter response times for these names due to an increased availability during recall.

Correct retrievals of recently learned names showed a significant positive correlation with perfusion increases in the right anterior hippocampus where (only in part) older items also led to significant though weaker correlation coefficients. By contrast, however, correct recent items were also significantly correlated with perfusion increases in dopaminergic midbrain areas comprising the ventral tegmental area and the right substantia nigra (VTA/SN complex). Correct retrievals of older names did not exhibit any significant correlation in this area.

Although involvement of midbrain regions should be treated with caution in BOLD fMRI, MR-based perfusion imaging with gradient echo acquisitions has been shown to substantially decrease the risk of susceptibility artefacts and signal drop-outs compared to BOLD images because a minimum time of echo (TE) is used [26]–[28], [16]. Reliability of this observation is further supported by recent fMRI studies on midbrain structures that also did not use higher spatial resolution than 64 by 64 in-plane pixels [29]–[31] and re-analysis of the data showed that the involvement of midbrain areas remained stable with a smaller smoothing kernel of six millimetres full width at half maximum (FWHM).

The observation that recent but not older correctly retrieved names were correlated with perfusion increases in dopaminergic midbrain regions is in line with previous reports of the dopaminergic influence on strengthening previously acquired labile new memories [29], [32]–[34]. Tracer studies (for review [35]) have demonstrated that the hippocampus receives dopaminergic projections from midbrain areas (ventral tegmental area and substantia nigra), and in vivo as well as in vitro electrophysiological studies support a specific neuromodulatory [36] role for dopamine in the temporal persistence of long-term potentiation [37]–[40], [32] which enhances the subsequent storage of information by the hippocampus once this information has been computed as novel at or around the time of encoding [41], [42].

Taken together, this dissociating pattern of correlations supports the idea that perfusion increases at least in the hippocampus and the left lateral temporal cortex are related to the off-line memory processing of previously learned face-name associations. The absence of correlations in the other regions with significant perfusion increases may be due to different reasons: Either the regressor in terms of correctly retrieved items was possibly not sensitive enough to produce significant correlations. Or these regions are indeed not functionally related to off-line memory processes but to some other mental processing for which we did not control experimentally.

### Limitations of the study design

Although the dissociating pattern of correlations supports that perfusion increases are related to off-line memory processing, future studies should include an active control condition (instead of no-learning) between Pre1 and Pre2 to further support the specificity of post-learning perfusion increases.

Another limitation of the present study design is reduced experimental control during perfusion scanning. We sought to investigate off-line memory processing as directly as possible and therefore perfusion scanning was performed without concomitant functional challenge to direct or control subjects' mental engagement after learning (e.g. [43]), or during sleep [44], [45]. However, intentional rehearsal of previously encoded face-name associations is unlikely to account for the rCBF changes observed for several reasons. First, at debriefing all participants reported the final recall task to have come as a surprise, since they had already judged the last selective reminding as the end of the experimental part of the session. Second, all participants denied rehearsal when explicitly asked whether they had engaged in such thinking. Third, rCBF activation patterns in the contrast of Post-Pre2 did not include known loci of activations for silent mental repetition [46], [47]. Especially areas activated by inner speech (Broca's area) [48], [49] did not emerge even at the very low significance level of p = 0.10.

It might also be argued that due to the temporal ordering of the experimental conditions a potential alertness confound could have biased the rCBF comparisons. Subjects experienced a rather long period of rest before the challenging encoding and selective reminding tasks which were again followed by a period of rest during the post perfusion scan. Therefore, the increase in rCBF during post learning perfusion compared to its preceding scans could reflect non-specific changes in alertness. An extreme case of this scenario could even be that some subjects felt asleep during Pre2 and that the rCBF increases in Post perfusion scanning would merely be correlated with the brain just have been woken up. However, analysis of heart rates as an approximation to monitor states of alertness of individual subjects across the different sessions of rest did not support that any of the subjects fell asleep or woke up from sleep during the experimental session. Therefore, it is unlikely that our data can be explained by substantial variations in subjects' overall state of alertness.

Finally, it is of note that in this first experimental approach only men were included which represents a limitation with respect to the generalizability of results.

### Non-intentional rumination as a candidate process?

Given that neither intentional rehearsal nor different states of alertness have contributed to the changes observed, it is still possible that non-intentional rumination occurred during Post perfusion scanning. This, however, does not represent a limitation we owe to the reduced experimental control. Instead these processes integrate with the proposal that after learning parts of the brain reveal patterns of neural activity resembling the activity patterns that had occurred during the corresponding experience (see Figure S3 of Supporting Information for results). Since this neural activity was differentially correlated with associative memory performance we assume that it could resemble the non-intentional reactivation of memory traces and not merely a neurophysiologic consequence of the intensive learning that had taken place before. Similarly, the pattern of significant correlations also contradicts the idea that reactivation simply represents epiphenomena of neural activities reflecting the subjects' thinking about preceding learning. Unfortunately, the limited temporal resolution of perfusion imaging did not permit more detailed insight into the hippocampal-neocortical interplay subserving memory trace reactivations as recently shown by a study of Tambini and colleagues [15]. In a very similar design to the present study, hippocampal-cortical correlations of spontaneous BOLD fluctuations during rest, before and after two different association tasks, were compared. These correlations were differentially enhanced depending on the level of later associative memory performance obtained from a memory test that followed post-encoding rest. As in the present study, individual differences in the magnitudes of post minus pre-learning hippocampal-cortical correlations also predicted individual differences in later associative memory. Therefore, these results provide further support that enhanced neural activity during rest after an associative encoding task is related to long-term memory performance.

### Conclusion

Using MR-based perfusion imaging as an absolute measure of neural resting state activity, present findings offer one of the first experimental steps towards the direct investigation of off-line processing of associative memories in the awake human brain. We propose that at least parts of a neural network were observed that may serve as an a priori region of interest in future studies in order to provide further evidence to support that reactivation of memory traces as one possible mechanism plays a crucial role in stabilizing long-term persistence of associative memories.

## Materials and Methods

### Subjects

The study was part of a larger memory project for which approval of the local ethical committee at the Ulm University Medical School has been obtained. Fourteen healthy, right-handed males (mean age: 24.4 years; SD: 2.95) with normal or corrected vision were included. All subjects provided written informed consent and none of the subjects had a history of neurological, psychiatric disorders, or was taking medication.

### Procedure

Prior to imaging, participants were told that they will have to perform two unrelated tasks and that imaging of task-free periods of quiet wakefulness will be performed. The session (Figure 1A) started with an anatomical MR scan in order to make participants familiar with the scanning situation. Thereafter, the first perfusion imaging was performed for 16 minutes (Pre1) that comprised two consecutive sub-blocks of 8 minutes. Separated by a break, during which no imaging or any other task occurred, a second 16-min perfusion imaging followed (Pre2). The duration of the intermediate task-free break was about 20 minutes and corresponded to the overall duration of initial learning and the consecutive selective reminding (SR) tasks (see below). Upon completion of the last SR block, perfusion imaging was started again with duration of 16 min (Post). As with the Pre perfusion imaging, participants were instructed to lie still and relax. During perfusion imaging, the video goggles were switched off such that participants saw an entirely black screen and no ambient light. At the end of the Post perfusion scan subjects were confronted with a surprise cued associative recall task (CR) to assess learning success. Upon completion of imaging, participants were debriefed with regard to intentional mental rehearsal during quiet wakefulness, and the extent to which the final recall was a surprise.

Throughout the entire experiment heart rate was measured in each subject as an approximation to his state of alertness across the different sessions of rest (Pre1, Pre2, and Post). Heart rates were re-sampled over intervals of 60 seconds each. In a recently published study [50] mean heart rates have been aligned with different sleep states (non-rapid eye movement sleep (NRMS) 1 and 2, and 3 and 4, as well as rapid eye movement sleep (RMS)). As our subjects could have been in any sleep state we calculated the mean heart rate (58.0 beats/minute) across NMRS and REM states as reference to compare with the heart rate associated with quiet wakefulness (65.2 beats/minute). The relative difference of 11.2% heart rate change was used as a cut-off score in order to individually test whether each of our subjects changed his state of alertness when comparing the start of Pre1 (second minute) against the end of Pre2 (last minute), or between the end of Pre2 and the entire time-course of the Post interval in bins of 60 seconds each.

### Experimental tasks

#### Encoding task

In order to assess the formation of declarative associative memories face-name associations were used as stimulus material. This material inherently requires the formation of associations and has already been shown to robustly involve hippocampal activation [51]–[54]. Also in the present study, fMRI was performed during the initial encoding of 24 face-name associations, but detailed presentation of this data is beyond the scope of the paper. It is of note, however, that we confirmed previous findings of strong involvement of the hippocampus which was reflected by a subsequent memory effect with graded neural activities depending on the success of an ensuing retrieval task (see Figure S3 of Supporting Information for results).

Each of the 24 face-name associations consisted of a coloured face centred on a grey background with a fictional first name printed in black colour underneath the face. Each face-name association comprised 7.35° in vertical and 5.6° in horizontal direction of the visual field. The face stimuli were portrait-style digital colour photographs of Caucasian, female faces assigned to four different groups of age (21–30 years, 31–40 years, 41–50 years and 51–60 years). Hair, ears and the part of the neck with the jugulum as the lowest border were left visible in order to simulate everyday encoding requirements. Images with rare and easily recognizable features such as scars, ear rings, glasses etc. were not used. For each age group first names were obtained from a website (www.beliebte-vornamen.de) listing commonly used German first names per decades that were assigned to the estimated age of the face to rule out sharp and possibly biasing contrasts between faces and names due to age effects (i.e. combining an “old” face with a “modern” name). The use of female faces only was due to technical restrictions of the name retrieval procedure. During cued associative recall the names had to be recalled using a 9-button device representing 9 different letters. Names were chosen such that they could be assembled from a fixed set of nine letters (*a-e-i-c-h-n-r-s-t*) on a customized response keyboard. For this task the frequency of appropriate German first names had a marked bias favouring female names, so that a balance between female and male names could not be achieved. Stimuli were presented via MRI compatible goggles (Resonance Technology, Inc.; Northridge, California) by means of commercial stimulation software (Presentation version 9.20; Neurobehavioral Systems, Inc.; Albany, California).

To ascertain that subjects processed each face-name association, they had been instructed to subjectively decide whether the name somehow “fitted” the face, or not. Decisions were made by button presses (left button: fit; right button: no fit). Each face-name association was presented for three seconds in a slow event-related fMRI design with an average stimulus-onset asynchrony of 25.8 seconds. During baseline a fixed, previously over-learned face-name association was shown. The color of the name randomly changed between blue and red. Upon each change, participants had to press keys under their right index (red) or middle finger (blue).

### Selective reminding

Initial encoding was followed by a selective reminding (SR) task [55] which was used to ensure a rather homogeneous amount of learned information across subjects of at least 40% of the associations of the initial set. During this task MR scanning was switched off and participants responded via a microphone incorporated in an MRI-compatible head set. During each SR block all faces from the initial encoding set were presented pseudo-randomly in permutated order to eliminate potential serial position effects [56]. Upon appearance of a face subjects were asked to recall the associated name. Whenever participants failed to correctly name the actual face or to give a response at all, the name belonging to this face was shown again for 15 s. Over the three SR blocks two types of correct responses could be classified: correct older and correct recent names. Correct older names were operationally defined as those names that had already been correctly retrieved at least once during one of the preceding SR blocks (e.g. a name correctly retrieved during the first SR block, but not during the second block and then again during the third block). Complementary, correct recent names were operationally defined as those names that had not been correctly retrieved during a preceding SR block, but had been just learned during a preceding SR block such that they could be correctly recalled during the actual SR block.

### Cued associative cued-recall (CR)

This task came as a surprise after the Post perfusion scan (Figure 1A). For understanding of the experimental parameters below it is of note that also this task was performed during self-paced slow event-related fMRI. However, report of the data is beyond the scope of the present paper.

Participants had to retrieve the names associated with the faces by means of a customized MRI compatible keyboard with a 3 by 3 button arrangement. Presentation order of faces serving as the cue was permuted with respect to the last selective reminding task (SR3). Assignment of letters to keys was displayed beneath the face (Figure 1 C). Participants could see this assignment and their input while typing. Two keyboard buttons had additional functions. Incorrect letters could be deleted with the lower left button pressed for more than 300 ms. Pressing the lower right button for this amount of time submitted the input. The face, the participant's input, and the key assignment remained on the screen until submission.

Participants were instructed to enter the letter combination *aaa* or *eee* when unable to guess a name within a reasonable time interval, or to recognize the face, respectively. Thus, four types of a response could be collected by the device: correct and false responses, and responses where participants could not find a name (*no name*), or could not recognize the face (*unfamiliar face*).

During retrieval, no external time constraint was imposed on the participants, although they were instructed to respond as fast and accurately as possible. As baseline stimulus always the same and well learned face was presented, along with one randomly chosen letter of its name. Upon appearance of the letter, participants had to press the corresponding key. Prior to recall, participants were trained to operate the keyboard which took about 5 minutes including instruction. All responses and time stamps were registered by a PC. After sending a completed response 18 to 21 s of rest elapsed. Mean duration of the recall task was 20.5 min (SD: ±1.4; range, 19.0–23.4 min). During recall, participants were not given any feedback on their performance. Like for the SR task correct responses could be classified into two response categories: older correct names, i.e. names that had already been correctly retrieved at least once during one of the three SR blocks, and recent correct names, i.e. names that had just been learned during the very last SR block (SR3) prior to Post perfusion scanning such that they could be correctly recalled during the final surprise CR task.

### Imaging data acquisition

Imaging was performed on a 3.0 Tesla head-only MR scanner (Siemens Magnetom Allegra; Siemens AG, Erlangen, Germany), using a standard transmit-receive head coil. High resolution T1-weighted anatomical images were obtained using a 3D MPRAGE sequence with 130 Hz/Px bandwidth, 2.3 s repetition time, 1.1 s inversion time, 3.93 ms echo time, 12° flip angle, and 256×256 (1 mm×1 mm) in-planar matrix resolution. At least 160 contiguous sagittal slices of 1 mm thickness were scanned, depending on brain size. Scan time lasted for about 8.5 minutes.

For continuous arterial spin labeling (CASL; see Wang et al., 2005 for further technical details) we used the same sequence as reported previously [57]. In brief, the labeling plane was 8 cm beneath the centre of the imaging sections. 20 radio-frequency (RF) pulses of 100 ms duration and a gap of 7.5 ms were used for labeling. A delay of 1 s between the end of the labeling pulse and image acquisition was inserted to reduce transit-related effects. Off-resonance artefacts were controlled by a sinusoidally amplitude-modulated version of the labeling pulse. T2*-weighted interleaved label and control images were acquired using a gradient echo echo-planar imaging (EPI) sequence (matrix 64×64 pixels, repetition time 4 s (including labelling and transit-time), echo time 16 ms, bandwidth 3005 Hz/Px). 18 transversal slices were positioned along the AC-PC line (thickness 5 mm, 1.5 mm gap). In-plane resolution was 3.44×3.44 mm. One perfusion scan (e.g. Pre1) comprised two consecutive sub-blocks, each with 120 acquisitions of labelled and control images. The duration of one sub-block was 8 minutes. Prior to each perfusion block potential changes of head position were assessed with a localizer scan. Whole-brain shimming routinely followed each of the localizer scans.

### Statistical analyses

#### Behavioral data analysis

To statistically compare rates of correct older and recent responses obtained by the various SR blocks and the final CR task an analysis of variance for repeated measures was computed in combination with additional post-hoc tests (Newman-Keuls; nominal level of alpha set to p<0.05). In all analyses of variance repeated measures were accounted for by Greenhouse-Geisser (GG) corrections (only GG-corrected p-values are reported in combination with the GG-epsilon to adjust degrees of freedom).

Response times for correct older and recent names in the CR task were compared using a t-test for paired samples. Individual response times for each trial were defined as the time interval that elapsed between presentation of the cue and the time point of the first key press associated with typing the entire correct name. Individual averages of response times for correct older and recent names were compared using a t-test for paired samples (level of significance: p<0.05).

### Imaging data analyses

Preprocessing and statistical analyses of perfusion data was very similar to a previous study [57] and was performed using Statistical Parametric Mapping (SPM5, Wellcome Department of Cognitive Neurology, London, UK; www.fil.ion.ucl.ac.uk) in combination with software implemented in MATLAB for use as a toolbox under SPM5. The toolbox code is based on a MATLAB script by H.Y. Rao and J.J. Wang from the Center for Functional Imaging at the University of Pennsylvania (http://cfn.upenn.edu/perfusion/software.html) that implements a single compartment continuous arterial spin labeling perfusion model (Wang et al., 2005) for reconstructing images of raw perfusion and quantified regional cerebral blood flow (rCBF) in units of mL/100 g tissue/minute.

Images of each sub-block underwent realignment to the first image to correct for head movements, reslicing, and generation of perfusion-weighted images by pair-wise subtraction of the label and control images, followed by conversion to quantified rCBF. This procedure also incorporated calculation of a mean EPI by averaging across all EPI images acquired during a single sub-block. Averages of sub-blocks 2–6 were co-registered onto the average of sub-block 1. The resulting transformation matrices were applied to all rCBF images of a sub-block, thereby aligning all rCBF images of the entire session. Next, individual T1 images were co-registered onto the average EPI image from sub-block 1. Normalisation of rCBF images into canonical MNI (Montreal Neurological Institute) space was achieved using the DARTEL process stream [58] as implemented within the current SPM8 (release 3684) software package. Normalized images were resliced to 2×2×2 mm^3^ voxels and finally smoothed in space with a three-dimensional 10 mm full-width at half-maximum (FWHM) Gaussian kernel.

In general linear models (GLM) for voxel-wise first-level rCBF analyses the time course of the volume mean was used as a covariate to reduce spatially coherent noise [27]. Regional CBF images were scaled to a grand mean of 50. No temporal filtering was involved. Within this first-level analysis mean CBF images were computed per each perfusion block. Images of contrast estimates were transferred to a group-level random effects analysis with factors Time (six levels, one for each sub-block from Pre1, Pre2, Post; Figure 1A) and Subjects in order to remove variability due to differences in the participants' average responses. Differences in mean rCBF in different contrasts (Post minus Pre2, Pre2 minus Pre1) were analysed by one-tailed *t* tests. Effects were considered significant at a statistical threshold of *p*<0.005 (uncorrected) at the voxel level and an extent threshold of 184 contiguously significant voxels to constitute clusters of significant rCBF differences at *p*<0.05.

Within the mask of significant rCBF differences between Post-Pre2 at the group level, additional regression analyses were computed to test for significant positive correlations between rCBF changes and the percentage of correct responses during the final associative cued recall task. Since the selective reminding task permitted separate calculation of older and recent correctly retrieved names two different regression analyses were run, one for each regressor (see Results section). Finally, in order to statistically test on a putatively dissociating pattern obtained from both regressors mean correlation coefficients were computed averaging across all within-mask voxels that already showed an association between changes in rCBF and either regressor. Therefore, an initial statistical threshold was set to p<0.05 during regression analyses. Heights of correlation coefficients were then statistically compared using a test for dependent correlations proposed by Steiger (see [59], p. 213; [60], formulas (14) and (3)). Critically, significant differences between two correlation coefficients were inferred at a nominal level of p<0.05, Bonferroni-corrected to account for multiple comparisons.

## Supporting Information

Figure S1 Positive significant correlations with either correct retrievals of older (blue) or recent (yellow) correct names. The region in the right anterior hippocampus color-coded in green shows voxels where perfusion increases were significantly correlated with both regressors.
(TIF)

Figure S2 Re-analysis of correlations between individual Post-Pre2 rCBF increases and individual numbers of correctly retrieved older and recent names (for calculation see Methods section in the main text). (A). Significant positive correlation with correct older names in the left middle temporal gyrus (MTG) and right hippocampal region comprising the right anterior hippocampus proper (HC) and parts of the anterior parahippocampal gyrus (PHC). (B). Significant positive correlation with correct recent names in the right anterior hippocampus (HC) medially extending into midbrain regions comprising the substantia nigra (SN). Scatterplots, correlation coefficients and associated p-values are from the peak voxel of each cluster.
(TIF)

Figure S3 **(A)**. Overlay of significant effects of encoding and Post-Pre2 perfusion increases in the left and right hippocampus on the group-averaged standardized T1-weighted brain image in MNI standardized space. The blue voxels bear the significant (p<0.05, family wise error correction) encoding effect averaged across trials of successful and unsuccessful encoding, and those trials where subjects were not in the position to make a response upon presenting the face during the cued recall task of the first SR block. Coded in purple color are hippocampal voxels that additionally showed significant increases of rCBF in the Post-Pre_2 contrast of perfusion imaging. **(B)** The bar charts reflect mean sizes of modelled effects averaged across subjects and significant blue voxels (error bars are standard error of the means). Depending on subjects' individual recall performances obtained during the first selective reminding block (SR_1) after initial encoding, graded neural activities were associated with successful encoding (operationally defined as Correct recall), unsuccessful encoding (False), and with encoding trials for which subjects could not give a response during the ensuing cued recall task (No Response).
(TIF)

Table S1 Brain regions with significant rCBF differences in the contrast Post-Pre2 after re-analyzing the fMRI data using a smaller smoothing kernel of 6 mm full width of half maximum during preprocessing. MNI: Montreal Neurological Institute; BA: Brodmann Area; Z scores of peak voxels refer to standard normal distribution and were derived from an one tailed t contrast on the difference in mean rCBF between the POST and PRE 2 perfusion scans with voxel-level significance of p<0.005, uncorrected, and cluster-level significance at p<0.05 (corresponding to 56 contiguously significant voxels). *: denotes anatomical locations with peak voxels also surviving a false discovery rate correction with p<0.05.
(PDF)
