# Supplementary figures and images for: Increased Resting-State Perfusion after Repeated Encoding Is Related to Later Retrieval of Declarative Associative Memories
Source: PLoS One. 2011 May 12;6(5):e19985. doi: 10.1371/journal.pone.0019985 (PMC3093410; doi:10.1371/journal.pone.0019985)

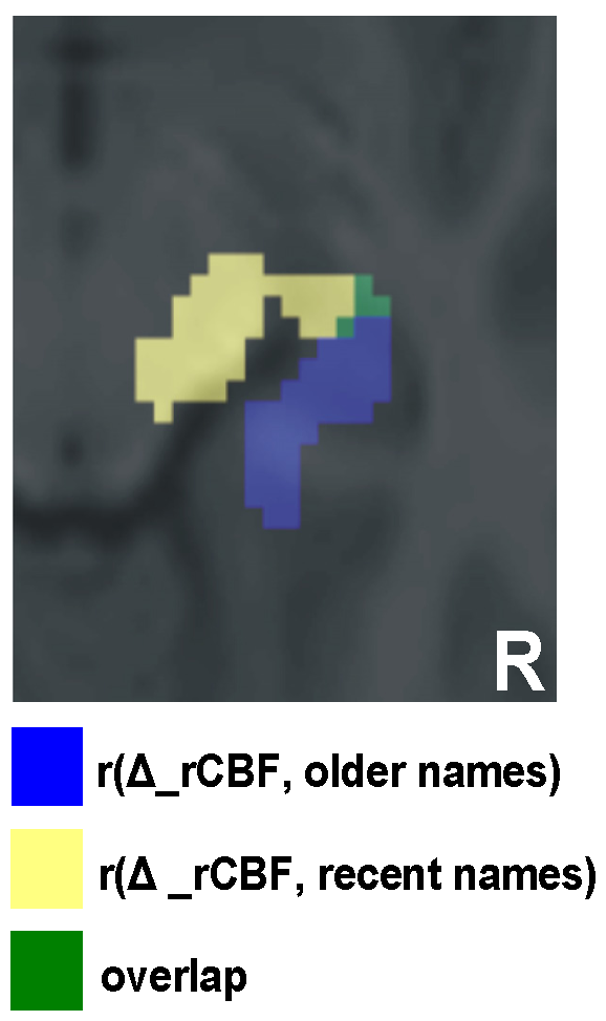

Supplement: Figure S1 — Positive significant correlations with either correct retrievals of older (blue) or recent (yellow) correct names. The region in the right anterior hippocampus color-coded in green shows voxels where perfusion increases were significantly correlated with both regressors. (TIF) [file pone.0019985.s001.tif]

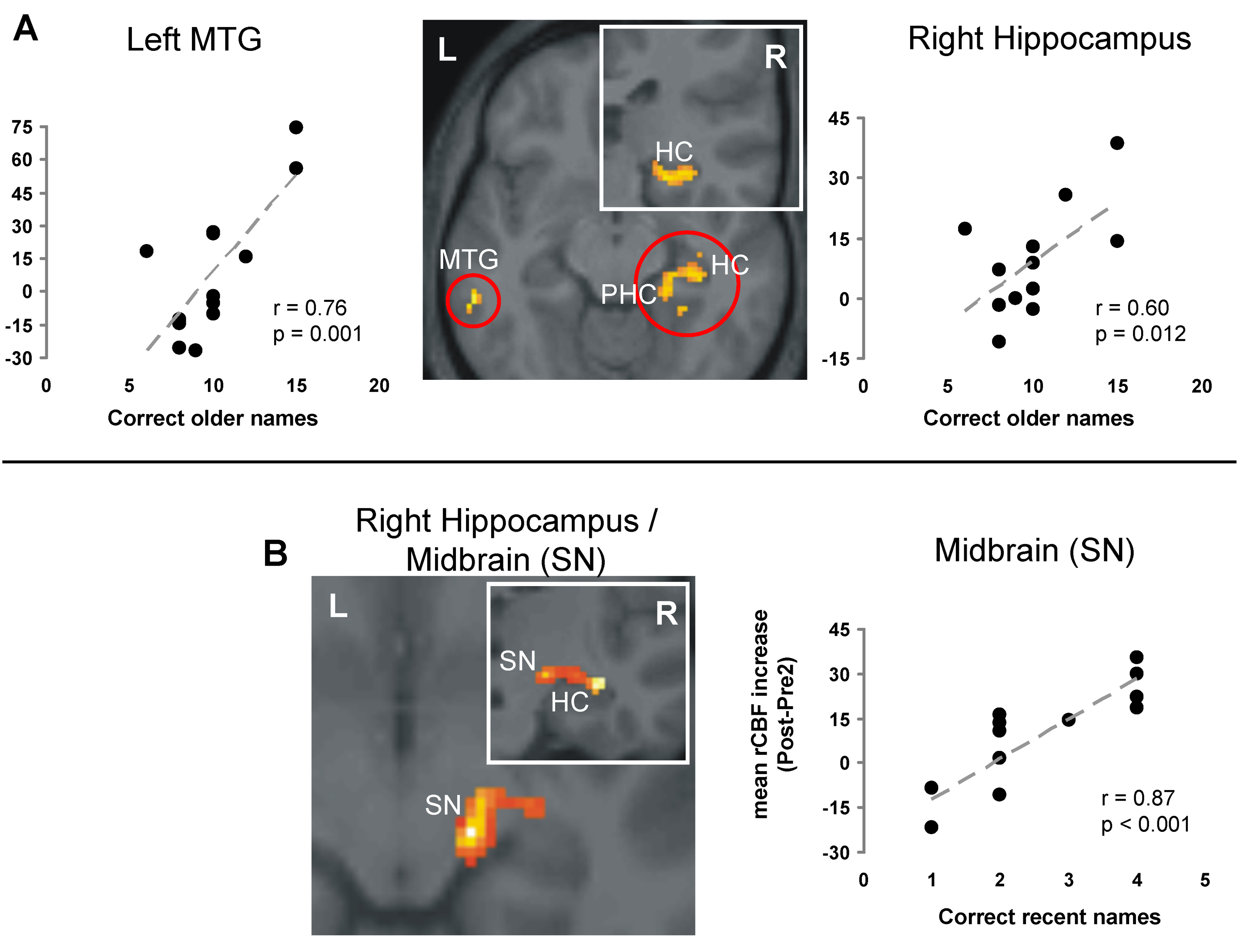

Supplement: Figure S2 — Re-analysis of correlations between individual Post-Pre2 rCBF increases and individual numbers of correctly retrieved older and recent names (for calculation see Methods section in the main text). (A). Significant positive correlation with correct older names in the left middle temporal gyrus (MTG) and right hippocampal region comprising the right anterior hippocampus proper (HC) and parts of the anterior parahippocampal gyrus (PHC). (B). Significant positive correlation with correct recent names in the right anterior hippocampus (HC) medially extending into midbrain regions comprising the substantia nigra (SN). Scatterplots, correlation coefficients and associated p-values are from the peak voxel of each cluster. (TIF) [file pone.0019985.s002.tif]

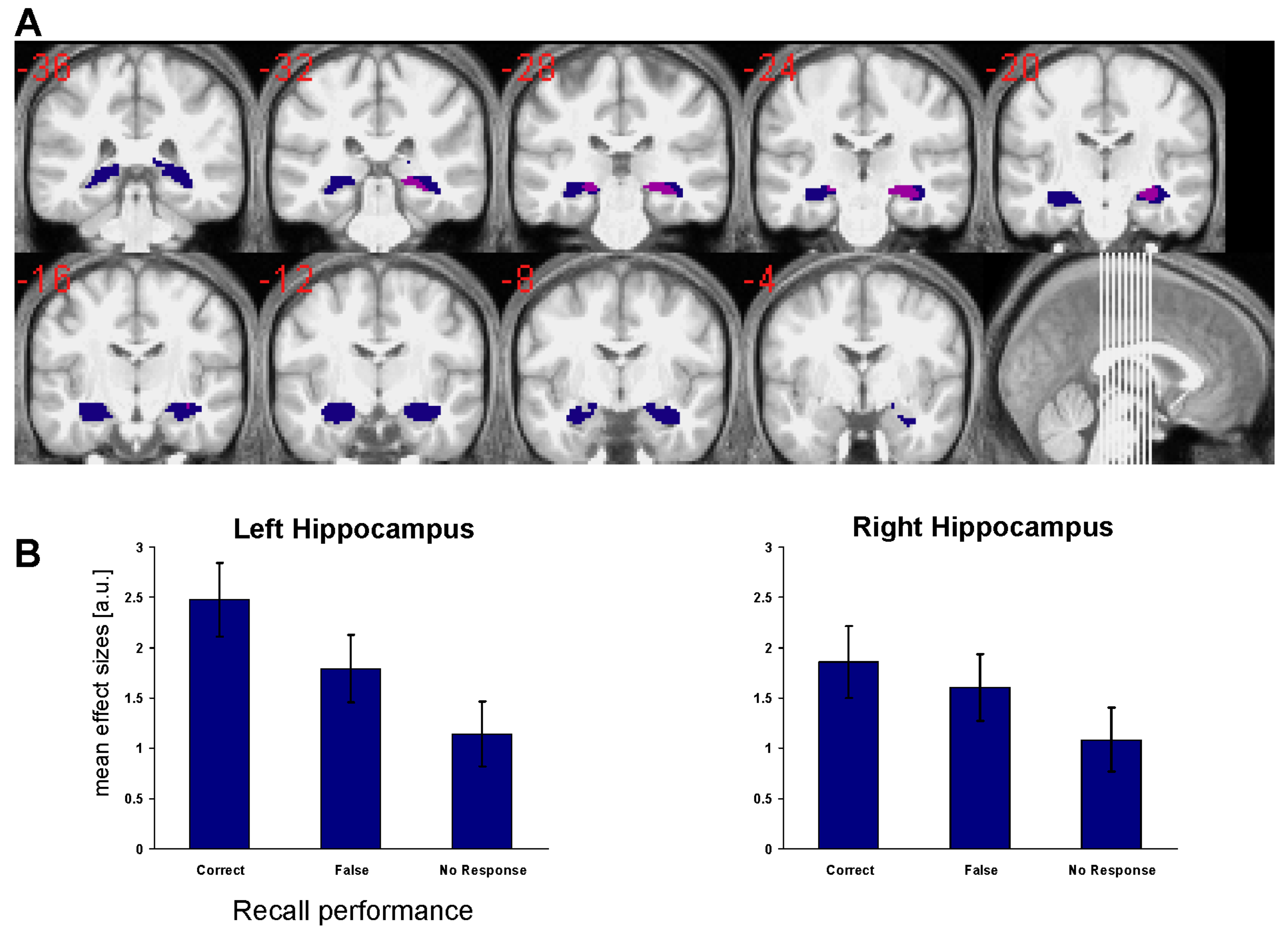

Supplement: Figure S3 — (A). Overlay of significant effects of encoding and Post-Pre2 perfusion increases in the left and right hippocampus on the group-averaged standardized T1-weighted brain image in MNI standardized space. The blue voxels bear the significant (p<0.05, family wise error correction) encoding effect averaged across trials of successful and unsuccessful encoding, and those trials where subjects were not in the position to make a response upon presenting the face during the cued recall task of the first SR block. Coded in purple color are hippocampal voxels that additionally showed significant increases of rCBF in the Post-Pre_2 contrast of perfusion imaging. (B) The bar charts reflect mean sizes of modelled effects averaged across subjects and significant blue voxels (error bars are standard error of the means). Depending on subjects' individual recall performances obtained during the first selective reminding block (SR_1) after initial encoding, graded neural activities were associated with successful encoding (operationally defined as Correct recall), unsuccessful encoding (False), and with encoding trials for which subjects could not give a response during the ensuing cued recall task (No Response). (TIF) [file pone.0019985.s003.tif]
