# Supplementary material for: Increased Resting-State Perfusion after Repeated Encoding Is Related to Later Retrieval of Declarative Associative Memories
Source: PLoS One. 2011 May 12;6(5):e19985. doi: 10.1371/journal.pone.0019985 (PMC3093410; doi:10.1371/journal.pone.0019985)
Supplement: Table S1 — Brain regions with significant rCBF differences in the contrast Post-Pre2 after re-analyzing the fMRI data using a smaller smoothing kernel of 6 mm full width of half maximum during preprocessing. MNI: Montreal Neurological Institute; BA: Brodmann Area; Z scores of peak voxels refer to standard normal distribution and were derived from an one tailed t contrast on the difference in mean rCBF between the POST and PRE 2 perfusion scans with voxel-level significance of p<0.005, uncorrected, and cluster-level significance at p<0.05 (corresponding to 56 contiguously significant voxels). *: denotes anatomical locations with peak voxels also surviving a false discovery rate correction with p<0.05. (PDF) [file pone.0019985.s004.pdf]

Table S1

Anatomical Region	***MNI coordinates (mm)***			***Z score***	***BA***
	x	***y***	***z***		
Right Hippocampus*	24	-28	-10	5.15	
Right Parahippocampal Gyrus*	26	-36	-10	4.44	36
Right Lingual Gyrus*	20	-44	-8	4.47	30
Midbrain (SN/VTA complex)	10	-20	-12	3.01	
Left Middle Temporal Gyrus*	-58	-30	-6	4.80	21
Left Temporal Pole*	-44	18	-28	4.40	38
Left Insula*	-36	12	-6	4.24	13
Left Anterior Cingulate Gyrus*	-6	30	20	4.27	24
Right Anterior Cingulate Gyrus	8	28	22	3.74	24
Left Hippocampus	-20	-30	-8	3.50	
Left Lingual Gyrus	-18	-42	-6	3.48	30
